# Chloro-Nitrous Oxyd

**Published:** 1869-02

**Authors:** J. C. Parker

**Affiliations:** Grand Rapids


### CHLORO-NITROUS OXYD.

BY DR. J. C. PARKER.

Read at the Michigan Dental Association.

GRAND RAPIDS, Oct. 12th, 1868.

*Mr. President and Gentlemen of the Society:*

In the subject of anæsthesia and anæsthetic agents we are all more or less directly interested, and to Dentists generally the question as to whát is the most safe and reliable anæsthetic agent, is one of great interest and importance to all.

Such being the fact, I shall make no apologies for calling your attention to the discovery of a new agent, which for want of a better name I shall designate as CHLORO-NITROUS OXYD. It is prepared by mixing with pure nitrous oxyd a certain portion of the vapor of chloroform during its manufacture. The quantity of chloroform used is comparatively small, the amount vaporized and mixed with sixty gallons of nitrous oxyd will probably not exceed one half a drachm.

During six months of its exclusive use by the discoverer, Dr. W. P. Barker, and a somewhat limited use by myself, chloro-nitrous oxyd has proved itself a safe, pleasant, and efficient agent, producing results satisfactory both to patient and operator. It is administered in the same manner as nitrous oxyd, the patient, however, being allowed to breathe more or less air as may be deemed necessary during its exhibition; the profundity and length of the anæsthetic state being governed very much by the skill of the operator in that respect.

If a short operation is to be performed, similar to extracting a single tooth, it is administered in the same manner as nitrous oxyd.

Its most marked effects upon the patient during its exhibition, are: 1st. Complete muscular relaxation; 2d. The quiet condition of the patient,—the most intractible, through fear or other causes, being under the control of the operator; 3d. The natural appearance of the countenance while under its influence; 4th. The anæsthesia being more profound, and the patient not being troubled by the peculiar vagaries and fancies of the imagination. There is a feeling of satisfaction produced in the mind of the patient, very gratifying both to the patient and operator; and while there is not the exhilarating effect often produced by nitrous oxyd, there is not the depression produced by chloroform.

I presume that all Dentists who have had much experience in the use of nitrous oxyd, have had patients declare after a successful and beautiful operation, that "they knew all about it," and felt more or less pain. Though this be purely imaginary on the part of the patient, and the impression will sometimes gradually pass off, it produces a feeling of dissatisfaction extremely annoying to the operator.

The quietness of the patient while under the influence of this agent, is probably due to the complete muscular relaxation, inducing a feeling of perfect ease, and causing the patient quietly and imperceptibly to pass into a state of complete anæsthesia.

The effect of this agent does not pass off as suddenly as does that of nitrous oxyd, the patient gradually returning to consciousness, but not so slowly as to interfere with the time of the operator.

Again, our experiences seem to indicate that the blood does not immediately flow as freely as when nitrous oxyd is used.

Another thing of interest to the profession is, that the same results can be obtained as heretofore, with about one-half the volume of gas formerly used.

I have thus briefly stated a few of the more important points of this agent, and should more information be required, it can be obtained by addressing the discoverer, Dr. W. P. Barker, of this city.

